# Solar-driven plasmon-enhanced photocatalysis: Co^2+^-doped ZnFe_2_O_4_ nanospheres-embedded ZnO nanosheets for effective degradation of dyes and antibiotics†

**DOI:** 10.1039/d4na00949e

**Published:** 2025-01-15

**Authors:** Antony Dasint Lopis, Karan Menon, K. S. Choudhari, Bhavana Kulkarni, Sanjeev P. Maradur, Suresh D. Kulkarni

**Affiliations:** a Department of Atomic and Molecular Physics, Manipal Academy of Higher Education Manipal Karnataka 576104 India suresh.dk@manipal.edu; b Materials Science & Catalysis Division, Poornaprajna Institute of Scientific Research (PPISR) Bidalur Post, Devanahalli Bengaluru Karnataka 562164 India

## Abstract

To ensure sustainable management and the availability of water and sanitation for all, a UN sustainable development goal (SDG) focused on promising methods to eliminate aqueous pollutants is urgently required. In this regard, solar photocatalysis, driven by freely available sunlight using low-cost, reusable photocatalysts, is a promising approach. In this context, we present a novel full-solar-spectrum photocatalyst with promising efficiency attributed to its laddered heterojunction and Ag-based plasmon enhanced activity. Specifically, it comprised Co^2+^-doped zinc-ferrite nanoparticles embedded on zinc oxide sheets that were later conformally coated with a small weight fraction (2.5%) of Ag under sunlight. The photocatalyst was optimized for different synthesis methods, post-synthesis temperatures, and different compositions with orange G as a model pollutant. Unlike previous reports, without any scavengers, the photocatalyst was effective for highly polluted water with a chemical oxygen demand (COD) of ∼740 ppm, eliminating 66% of it within an hour. We have coined a new term, solar photo-oxidation efficiency (SPOE), to describe the photocatalyst's performance. SPOE was directly dependent on the pollutant concentration and was found to be 72% for 400 ppm ciprofloxacin, with an apparent quantum yield of 36%. The promising activity of our photocatalyst continued even after several reuses. The generation of hydroxyl and superoxide radicals was confirmed by respective confirmatory tests. Scavenging tests indicated the highest contribution of superoxide radicals and holes in photodegradation. Our photocatalyst is promising and holds enormous potential for use in the treatment of diverse pollutants.

## Abstract 2

A novel full-solar-spectrum photocatalyst with promising efficiency attributed to its laddered heterojunction and Ag-based plasmon enhanced activity for the decontamination of water from diverse pollutants.

## Graphical abstract

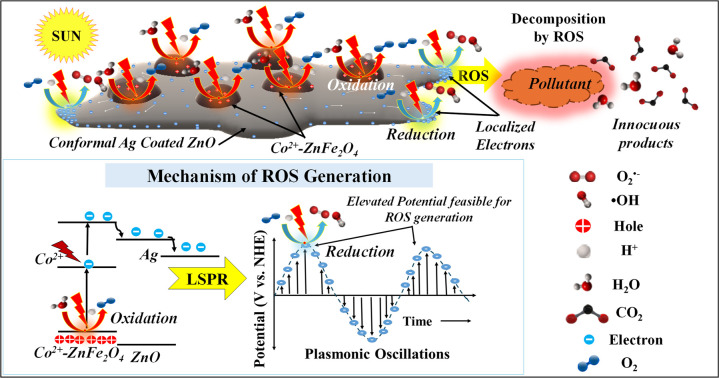

## Introduction

The United Nations proposed the sustainable development goals (SDGs) in 2015 with the aim of safeguarding the environment and advancing human civilization. The analysis has shown that wastewater treatment could contribute to 11 out of 17 SDGs.^1^ It is also clear that for sustainable wastewater treatment, the utilization of renewable energy resources is essential. As a renewable energy resource, freely available solar energy can be harnessed and converted into a desired energy form to achieve these goals, wherein photocatalysis is a prospective way to transform solar energy into suitable chemical energy for the remediation of polluted water.^2^

The semiconductor photocatalysis has been widely studied to photodegrade organic pollutants in water,^3–5^ as well as in air purification,^6^ hydrogen fuel generation,^7–9^ and antibacterial disinfection.^6^ However, developing a photocatalyst with high solar efficiency and resolving the global pollution and energy crises using a free and abundant energy resource (sunlight) remain challenging. Undoubtedly, the low efficiency of current photocatalysts stems from (i) the inability of the photocatalysts to use all wavelengths of sunlight and (ii) the rapid recombination of electron–hole pairs before they take part in photocatalysis.^10–12^ Efforts to improve photocatalytic efficiency in the past few decades include doping^13^ and the formation of heterojunctions,^5,14–17^ more specifically type-II heterojunctions,^18,19^ Z-schemes,^20–22^ S-schemes,^23–26^ plasmon-based enhancements,^27,28^ and laddered heterojunctions.^29–31^ Despite several efforts, we are yet to achieve a commercially viable solar efficiency of 5–10% for its commercialization,^12,32^ and achieving significant solar efficiency remains a challenge.^10^

Very recently, it has been shown that a laddered type-1 heterojunction formed between Fe^2+^-doped ZnFe_2_O_4_ and ZnO, without the use of scavengers, can harvest the entire solar spectrum for photocatalysis.^30^ Later, it was also revealed that a heterojunction formed by Co^2+^-doped ZnFe_2_O_4_ and ZnO also formed a laddered heterojunction capable of harvesting the broad spectrum of sunlight.^29^ However, the challenges now are to improve the solar efficiency by suppressing the charge-carrier recombination that is responsible for low efficiency. Hence, there is still a chance for laddered heterojunctions to achieve higher solar efficiency by eliminating the defects in the photocatalyst that cause recombination, as well as by forming a Schottky junction *via* selective conformal Ag-deposition on ZnO of the heterojunction, and to achieve an elevated reduction potential of photogenerated electrons by means of localized surface plasmon resonance.^31^

According to a previous report, synthesized Fe^2+^-doped ZnFe_2_O_4_ possessed a high amount of oxygen vacancies that were proven to be responsible for its lower photocatalytic activity.^30^ Annealing studies on Fe^2+^-doped ZnFe_2_O_4_ demonstrated its better activity when lowering the defects, but reducing them to a lower number was not possible, because the Fe^2+^ concentration dropped after annealing beyond 120 °C.^30^ Unlike Fe^2+^-doped ZnFe_2_O_4_, Co^2+^-doped ZnFe_2_O_4_ could be easily obtained with minimum defects (oxygen vacancies) by post-synthesis annealing far beyond 120 °C (400 °C for 4 h).^29^ The task now is to refine the synthesis in order to couple Co^2+^-doped ZnFe_2_O_4_ to a defect-free ZnO (as defects in ZnO can account for lower photocatalytic activity^33^).

This report illustrates two synthesis approaches, namely co-precipitation and microwave-assisted reflux methods, to optimize the photocatalytic activity of the Co^2+^-doped ZnFe_2_O_4_/ZnO composite. The defects of ZnO in the composite were minimized by post-synthesis annealing to achieve excellent photocatalytic activity under sunlight. The composite was conformally coated with Ag selectively on ZnO by the photocatalytic reduction of Ag^+^ under sunlight. The composites were then characterized using XRD, FESEM, BET surface analysis, and diffuse reflectance spectroscopy, *etc.* The photocatalytic performances of the composites synthesized by the two methods were evaluated under direct sunlight using orange G as a model pollutant. The effects of the annealing temperature, Ag-coating content, and ZnO content on the photocatalytic activity were investigated for its optimization. The optimized photocatalyst was further used to degrade ciprofloxacin and highly concentrated pharmaceutical solutions. To date, there is no method available to determine the photo-oxidation efficiency of a photocatalyst. Herein we introduce the term solar photo-oxidation efficiency (SPOE), based on COD measurements. Corresponding confirmatory and scavenging experiments were used to examine the ROS production during photocatalysis. A 5-cycle reusability test was performed on the optimized silver-coated composite. A photocatalytic mechanism based on a laddered type-1 heterojunction and plasmon-based enhancement is proposed.

## Results and discussions

**Fig. 1:**
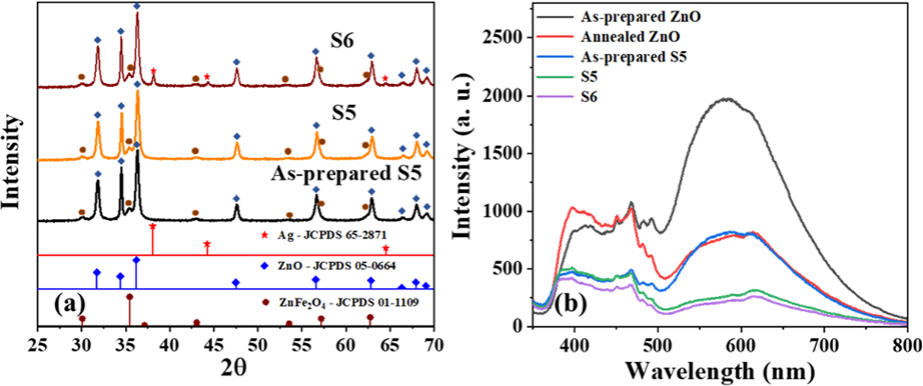
(a) XRD patterns of as-prepared S5, S5, and S6, which matched the standard JCPDS cards of Ag, ZnO, and ZnFe_2_O_4_, (b) photoluminescence spectra of as-prepared ZnO, ZnO annealed at 350 °C for 4 h, and as-prepared S5, S5, and S6.

Unlike the co-precipitation method (Fig. S1†), sample S5 obtained by the microwave-assisted reflux method was crystalline in its as-prepared form (Fig. 1a), and the peaks matched well with the respective JCPDS files corresponding to the cubic spinel crystal phases of ZnFe_2_O_4_ (JCPDS 01-1109) and ZnO (JCPDS 05-0664), and no other extra peaks were observed, implying the phase purity.^14,34^ The crystallite size, as determined using the Debye–Scherrer equation, showed a slight increase from 23 nm to 24 nm after 4 h annealing at 350 °C (S5). The XRD patterns of S6 (*i.e.*, S5 after Ag deposition) exhibited a few additional peaks with a relatively weak intensity that matched JCPDS file 65-2871, indicating the formation of FCC crystal-structured metallic silver.

The photoluminescence spectra of the as-prepared ZnO excited at 325 nm (Fig. 1b) showed substantial emission in the range of 500–800 nm, attributed to oxygen vacancies.^35,36^ The emission was quenched in this region after annealing ZnO to 350 °C for 4 h, indicating the healing of oxygen vacancies as a result of the heat treatment in the air (oxygen-rich environment). A similar result was found for sample S5, where the emission intensity corresponding to oxygen vacancies was significantly lower compared to that of the as-prepared form. This indicated that the ZnO in sample S5 possessed a low number of oxygen vacancies, and their low concentration is beneficial for excellent photocatalytic activity. Additionally, sample S6 showed a relatively lower emission intensity than S5, implying that the Ag on ZnO reduced the electron–hole pair recombination *via* the formation of a Schottky junction.^31^

**Fig. 2:**
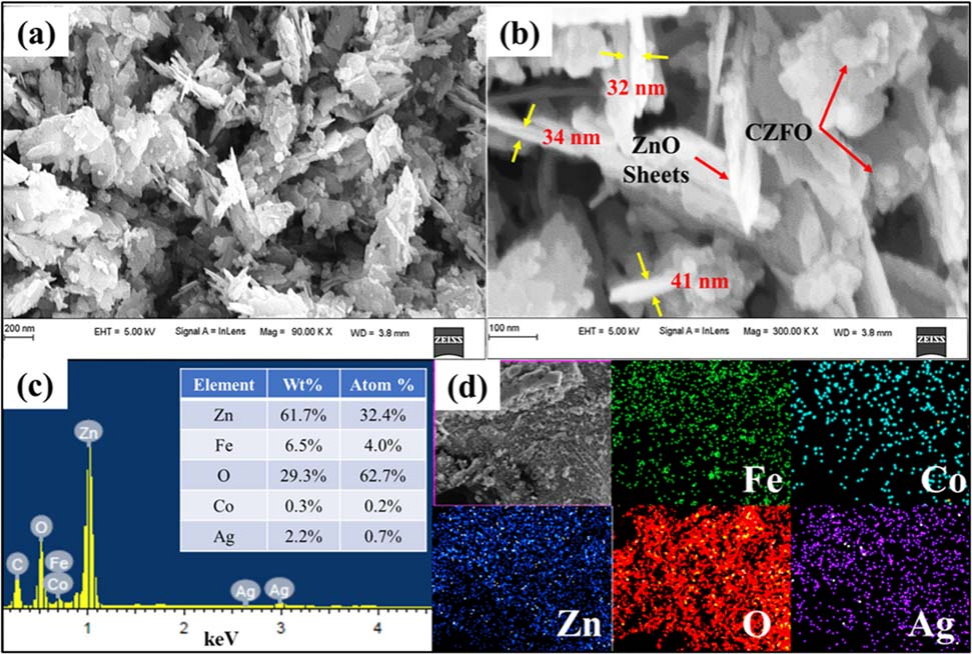
FESEM images of S6 at (a) 90 KX magnification, (b) 300 KX magnification, (c) EDS spectra; inset table shows the elemental composition, and (d) elemental mapping of Fe, Co, Zn, O, and Ag.

As evident from the FESEM, sample S6 was found to have a sheet-like structure (Fig. 2a) with sharp edges, which is crucial for enhancing the electric field and, therefore, the photocatalytic activity. CZFO nanoparticles, with an average size between 20 and 40 nm, were visible on the surface of the sheet (Fig. 2b). The sheet's thickness, determined from the enlarged image (Fig. 2b), was found to be between 30 and 40 nm, while the other two dimensions ranged from 100 to 400 nm. Inferring the purity of the composite, the EDS spectra (Fig. 2c) only displayed peaks for the elements Zn, Fe, Co, Ag, and O, except for the C from the carbon tape. By weight percentage, the elements Zn, Fe, Co, Ag, and O made up respectively 61.7%, 6.5%, 0.3%, 2.2%, and 29.3%. The weight percentage of silver detected (2.2%) was very close to the amount of silver used for the synthesis (2.5%), indicating the method was effective for achieving a good yield of Ag. The elemental mapping indicated the consistent distribution of Zn, Fe, Co, Ag, and O across sample S.

**Fig. 3:**
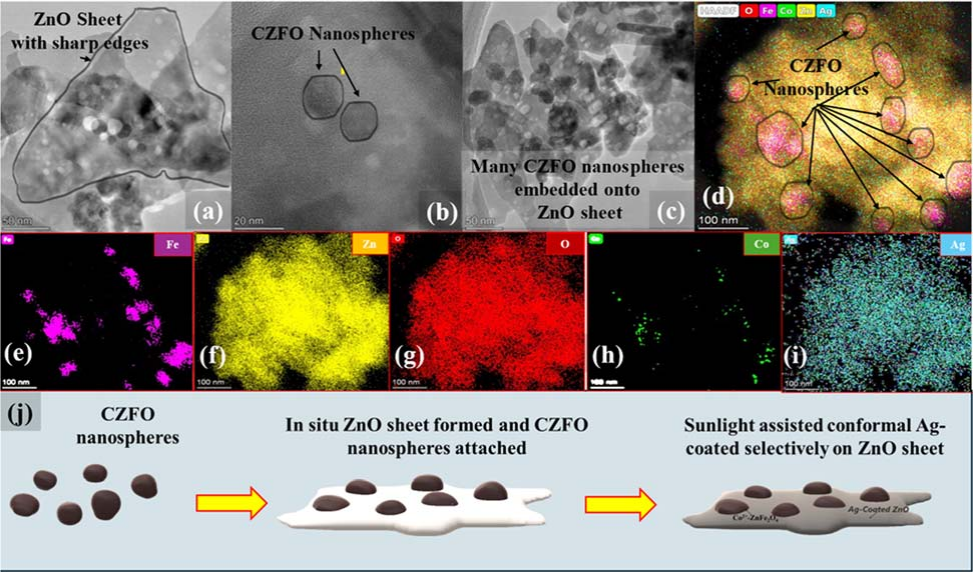
STEM images of S6, (a) ZnO nanosheet, (b) Co^2+^-doped zinc ferrite nanospheres embedded on the ZnO nanosheet, (c) multiple Co^2+^-doped zinc ferrite nanospheres embedded on ZnO nanosheets. (d) High-resolution elemental mapping on S6, illustrating the embedded Co^2+^-doped zinc ferrite nanospheres on the ZnO nanosheet conformally coated with Ag. Elemental mapping of (e) Fe, (f) Zn, (g) O, (h) Co, and (i) Ag (j) mechanism of S6 formation.

In agreement with the FESEM images (Fig. 2a and b) the scanning transmission electron microscopy (STEM) images of S6 (Fig. 3a–c) showed sheet-like structures of the sample with embedded CZFO nanospheres. The EDS spectrum (Fig. S2†) confirmed the presence of Zn, Co, Fe, O, and Ag in sample S6. The sheet-like structure of ZnO with embedded CZFO spherical nanoparticles was confirmed using high-angle annular dark-field imaging (HAADF). As shown in Fig. 3d–i, the elements Fe and Co were densely packed in the region that formed the morphology of CZFO. The presence of Zn and O throughout the sheet indicated that it was entirely composed of ZnO. Additionally, the distribution of Ag throughout the sheet indicated the conformal layer of silver on the ZnO sheet. Thus it was confirmed at the microscopic level that S6 was made up of ZnO sheets that were conformally coated with Ag and embedded with CZFO nanoparticles. Based on these observations, it could be inferred that (as shown in Fig. 3j) Co^2+^-doped ZnFe_2_O_4_ nanospheres were embedded on the ZnO sheet simultaneously during its formation, and later under sunlight it was conformally coated with Ag. The sharp edges of the ZnO sheets are helpful for plasmon-based enhancement in the photocatalytic activity.

**Fig. 4:**
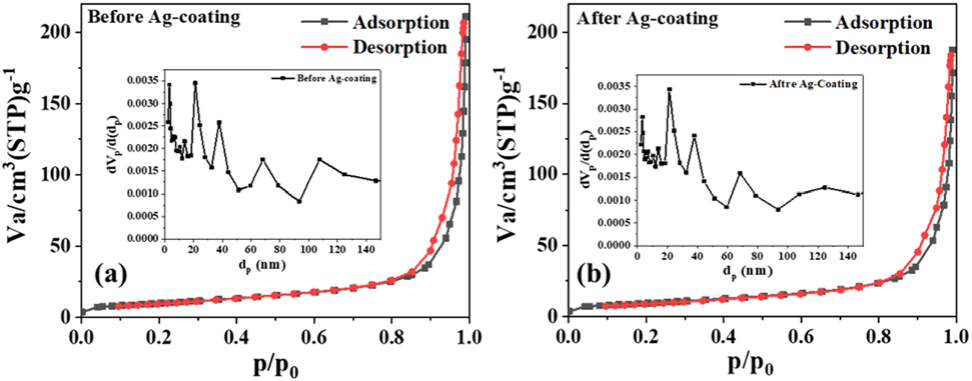
(a) N_2_ adsorption–desorption isotherms for S5 and (b) S6; insets show the respective BJH plots.

The N_2_ adsorption–desorption-based surface study (Fig. 4) revealed similar surface characteristics before and after the deposition of Ag, suggesting a probable conformal deposition of silver on S5. The mean pore diameter and volume slightly changed from 35 to 34 nm and 0.30 to 0.28 cm^3^ g^−1^, respectively, while the specific surface area was marginally reduced from 35 to 34 m^2^ g^−1^. After the Ag deposition, the slight increase in the size of the composite sheets may have caused a slight change in these parameters (Table S1†). The reduced average pore diameters by 1 nm suggested that the thickness of the Ag film inside the pores was 0.5 nm, indicating a probable monolayer Ag deposition on ZnO nanosheets.

**Fig. 5:**
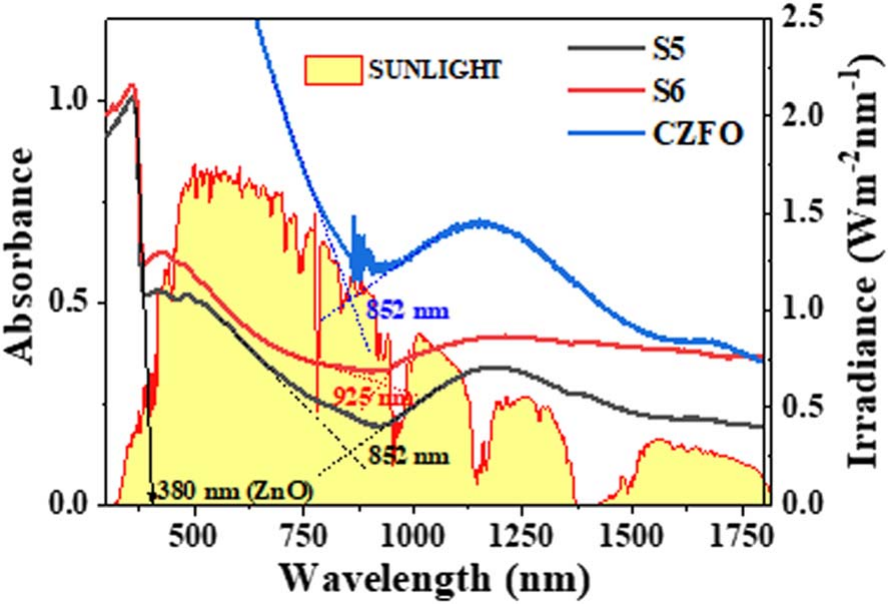
Absorbance spectra of S5, S6, and CZFO obtained from diffuse reflectance spectroscopy compared with the solar irradiance spectrum.

In order for a photocatalyst to function effectively under sunlight, its absorption spectrum must overlap with the maximum region of the solar spectrum. The absorbance spectra of S5, S6, and CZFO showed absorption from ultraviolet to near-infrared light in the solar spectrum on the Earth's surface (Fig. 5). The absorption edge corresponding to the excitation of valence band electrons for CZFO and S5 was at 852 nm. After the silver deposition (S6), this was redshifted to 925 nm. This redshift in the absorption edge occurred due to the plasmonic effect of the deposited silver in S6, making the photocatalyst S6 well-suited to efficiently harvest the entire region of sunlight for photocatalytic pollutant decomposition.

### Photodegradation studies under direct sunlight

**Fig. 6:**
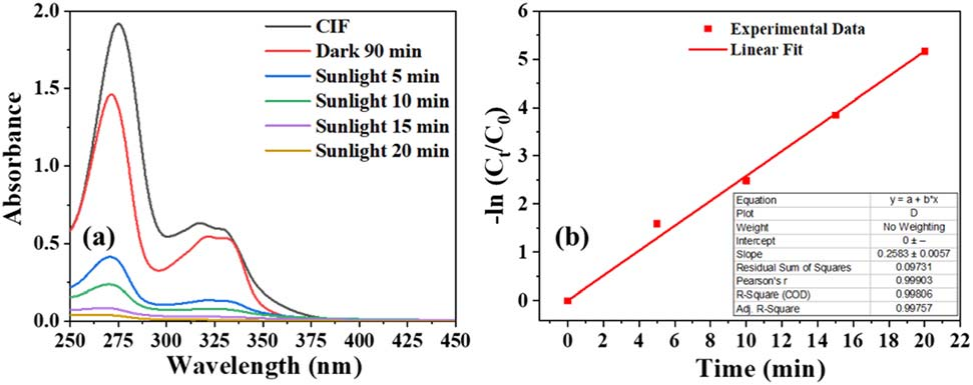
(a) Absorbance spectra of ciprofloxacin at different intervals during the photocatalysis, (b) respective apparent first-order kinetic plot, inset shows the linear fit parameters of the degradation kinetics.

The optimization studies of the photocatalyst for optimizing its composition, method of synthesis, and post-synthesis annealing are detailed in the ESI.† The optimized photocatalyst S6 was used to decompose ciprofloxacin, as a model pharmaceutical pollutant commonly found in the output of conventional wastewater treatment plants.^37^ Ciprofloxacin, which is transparent to sunlight, could be decomposed impressively in the presence of S6, with 80% of it eliminated within 5 min of exposure (Fig. 6). Complete mineralization was observed within 20 min of sunlight exposure. The apparent first-order rate constant was determined to be 0.258 min^−1^, which was 2.3 times higher than the observed rate constant for orange G.

### Treatment of high-COD pollutants

To test the effectiveness of S6 in decontaminating wastewater with a high chemical oxygen demand (COD), ciprofloxacin solution with a concentration of approximately 400 ppm was used as a representative pollutant. In this context, COD measures the amount of oxygen (in ppm) required to chemically oxidize organic pollutants. The standard closed reflux colorimetric method (APHA 5220 D) was employed to determine the COD of the ciprofloxacin solution at specific intervals. The degradation of ciprofloxacin was tracked by observing the decrease in COD values over time.

**Fig. 7:**
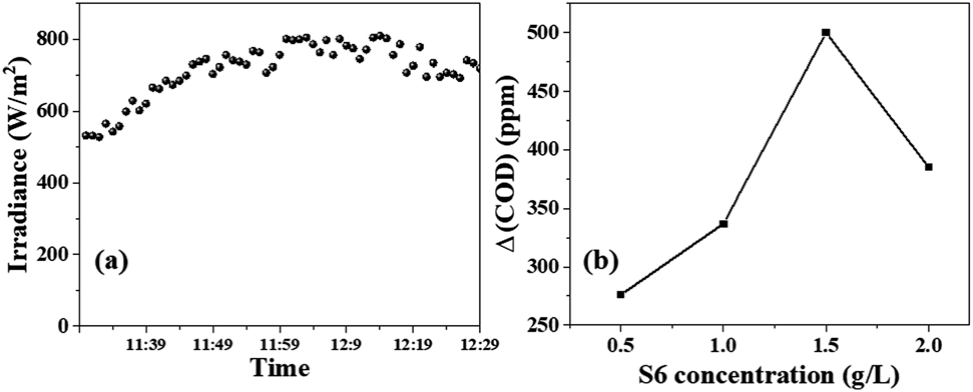
(a) Solar irradiance measured during the photocatalysis from 11:30 am to 12:30 pm, plot of (b) change in the COD of ciprofloxacin solution *versus* S6 concentration under 60 min sunlight exposure.

The solar irradiance was measured every minute for a total of 60 min, and the results are shown in Fig. 7a. The average intensity was found to be 740 W m^−2^. Fig. 6b shows that the ΔCOD during the 60 min sunlight exposure was dependent on the concentration of S6. The ΔCOD increased with the increase in the S6 concentration up to 1.5 g L^−1^, while it decreased with further increasing the concentration, indicating that 1.5 g L^−1^ was the optimum concentration. Therefore, S6 at the optimum concentration of 1.5 g L^−1^ was used to study Δ(COD) at different time intervals (Table 1).

**Table 1:** Variation of the solar photo-oxidation efficiency (SPOE) at different intervals of sunlight exposure

Time of exposure (*t* min)	Δ(COD) (g L^−1^)	No. of oxidated charges 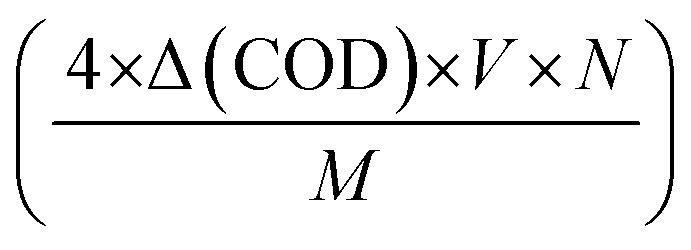	No. of incident photons *I* × *A* × *t*	Apparent quantum yield (AQY)	SPOE (%)
2.5	0.146	0.5495 × 10^21^	1.91853 × 10^21^	28.6%	57.3
5	0.204	0.7678 × 10^21^	3.82347 × 10^21^	20.0%	40.0
10	0.238	0.8957× 10^21^	7.86544 × 10^21^	11.5%	23.0
20	0.279	1.0576 × 10^21^	15.9647 × 10^21^	6.5%	13.1
30	0.308	1.1592 × 10^21^	24.2659 × 10^21^	4.8%	9.6

### Reusability test

Given the enormous amounts of pollutants produced by industries, the photocatalyst should preferably be inexpensive and capable of being reused multiple times without losing its effectiveness. To test this, photocatalyst S6 was extracted from the degraded solution and reused to decompose a fresh dye solution. It was observed that the percentage of degradation did not change significantly after five cycles of reuse, with only a 2% decrease in activity (Fig. S9b†). This suggests that the photocatalyst can be potentially reused several times.

**Fig. 8:**
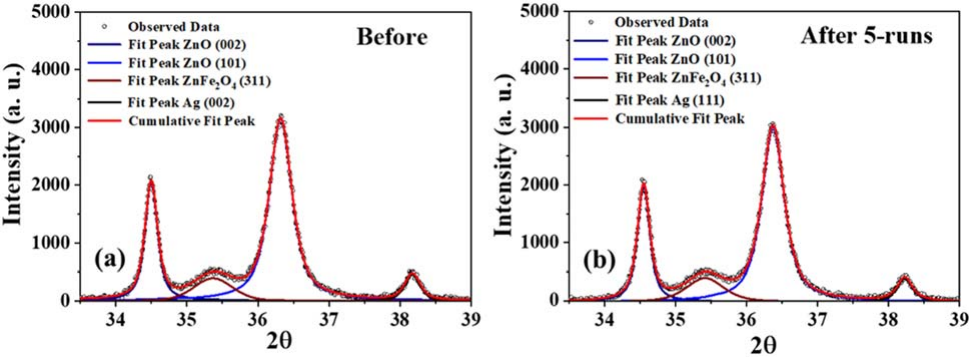
Deconvoluted XRD patterns of S6 (a) before and (b) after five successive photocatalytic runs.

Deconvolution of the patterns before and after 5 runs of photocatalytic degradation showed no significant difference in the relative peak area ratios of ZnO and ZnFe_2_O_4_, as well as for Ag and ZnO (Fig. 8). This means that there was no change in the crystal composition of S6, indicating that S6 remained intact even after five cycles of reuse. This demonstrates that S6 is a potentially reusable photocatalyst that could be used repeatedly to treat large volumes of effluents (Table S4†).

#### Determination of the solar photo-oxidation efficiency (SPOE) and apparent quantum yield (AQY)

We introduce a new parameter called the solar photo-oxidation efficiency (SPOE) to measure the solar efficiency of S6 for pollutant decomposition by converting sunlight photons into chemical energy in the form of ROS. The SPOE represents the percentage of solar photons used by photocatalyst to break down organic pollutants. It is the ratio of the number of photo-oxidated charges in the pollutant during decomposition under sunlight to the number of photons incident within a given time frame of exposure. To determine the number of photo-oxidated charges, the change in COD (ΔCOD) during the photodegradation can be used. COD is a measure of the amount of oxygen (in ppm) consumed during the oxidation of the organic pollutants. The ΔCOD value (in g L^−1^) can be used to determine the number of moles of O_2_ involved in the photodegradation process.

By multiplying this value by a factor of four (as molecular oxygen corresponds to the oxidation of four electronic charges), one can calculate the moles of photo-oxidated charges per litre. To determine the number of photo-oxidated charges during the degradation, we simply multiplied the volume of the solution in litres by Avogadro's number. Similarly, the number of solar photons incident on S6 can be calculated using the equation *I* × *A* × *t*, in which the photon density (*I*) (in number of photons per m^2^ per s) can be obtained by dividing the power density (in W m^−2^) by the average photon energy (in J). Hence, the expression for the SPOE can be calculated using eqn (1):1
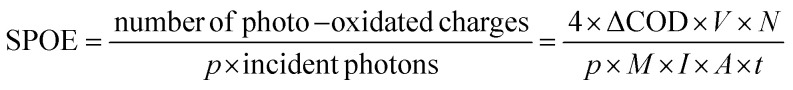
where ΔCOD represents the difference in COD values before and after sunlight exposure, *V* is the volume of the sample pollutant solution, *N* is Avogadro's number (6.02 × 10^23^), *M* is the molecular weight of O_2_, *I* is the incident photon density, *A* is the area of sunlight exposure, *t* is the time of exposure, and *p* is a factor depending on the photocatalytic mechanism. For a two-photon-assisted laddered heterojunction photocatalyst in which hydroxyl radicals are not generated from holes, the value of ‘*p*’ is 0.5. Essentially, ‘*p*’ represents the possible number of ROS generated per incident photon.

Similarly, the apparent quantum yield (AQY) can be calculated using eqn (2),2
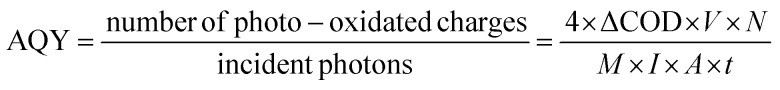


**Fig. 9:**
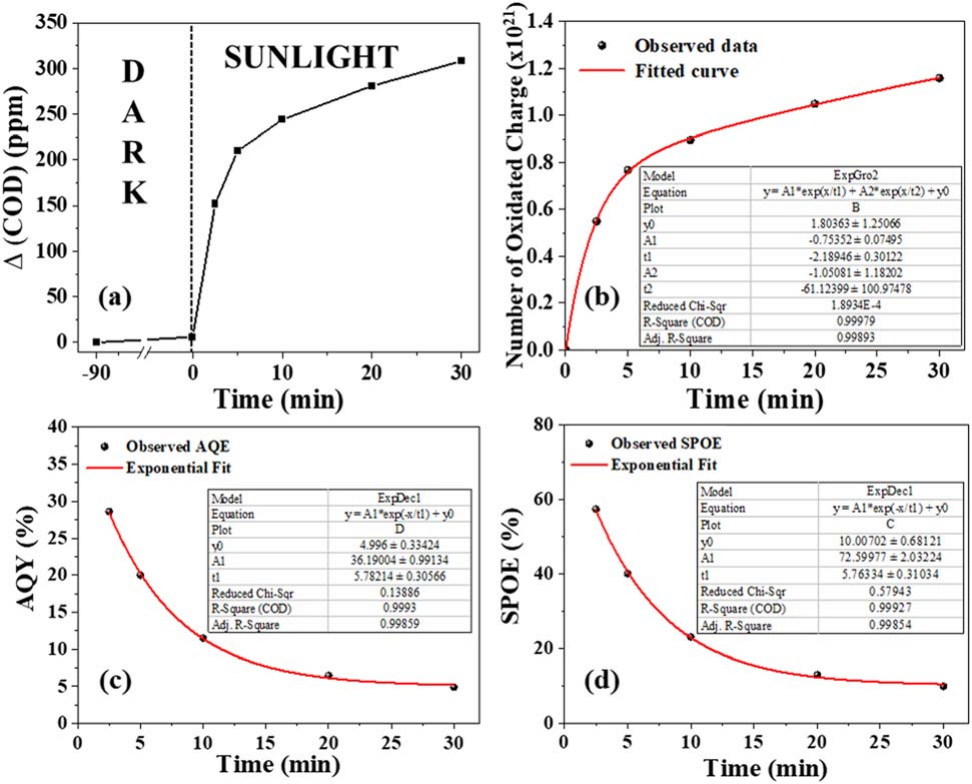
(a) Δ(COD) in the dark and at different intervals under sunlight, (b) number of oxidated charges *versus* time, (c) AQY and (d) SPOE at different intervals of sunlight exposure. The insets of respective images (b)–(d) illustrate curve fitting parameters.

The ΔCOD was measured at different time intervals (Fig. 9a) during photodegradation between 11:45 am to 12:15 pm when the irradiance remained nearly constant (Fig. 7a). The number of oxidized charges in the given interval increased exponentially (Fig. 9b), following two different time constants of 2.2 minutes and 61.1 minutes. This suggests that solar photooxidation is influenced by two distinct processes, consistent with the proposed mechanism in the later section. The process with a low time constant of 2.2 minutes can be attributed to the direct oxidation of ciprofloxacin by the photogenerated holes (or ROS), while the process with a longer time constant is linked to the oxidation by ROS (or holes). The plots of SPOE and AQY over time followed an exponential decay curve with decay constants of 5.76 and 5.78 minutes, respectively (Fig. 9c and d). The fittings of the curve indicate that the photocatalyst initially provides an impressive SPOE and AQY of 72% and 36%, respectively, when exposed to sunlight at maximum ciprofloxacin concentration (Table 1). The exponential trends in the SPOE and AQY implied the dependence of the solar-photo-oxidation on the pollutant concentration, following the law of mass action. This means that a higher pollutant concentration will lead to greater photo-oxidation due to the availability of more pollutant molecules in the vicinity of the photogenerated holes or ROS.

### Mechanism of the highly efficient photocatalytic degradation over S6

The photocatalytic mechanism of a semiconductor heterojunction photocatalyst is primarily grounded on the type of heterojunction, which enables the separation of electron–hole pairs generated by the light. To understand the type of heterojunction formed in the composite, it is crucial to know the band potentials of different components of the photocatalyst. According to a previous report,^29^ it was stated that the conduction band minima (CBM) of ZnO is −0.50 V, which is more negative than the CBM of CZFO (+0.14 V). Additionally, the valence band maxima (VBM) of ZnO is +2.7 V, which is more positive than the VBM of CZFO, at +1.59 V. Based on this, the CZFO/ZnO composite formed a type-1 heterojunction. However, the presence of ground-state conduction band electrons by the presence of Co^2+^ leads to a laddered type-1 heterojunction between CZFO and ZnO.^29^ When Ag was preferentially deposited on ZnO, electrons flowed from Ag to ZnO to equalize the Fermi level throughout both materials, resulting in the formation of a Schottky junction. This was due to the higher Fermi level of Ag (5.2 eV) compared to ZnO (4.26 eV).

The confirmatory test for ROS implied the generation of hydroxyl and superoxide radicals during the photocatalytic process (Fig. S12b†). Meanwhile, the scavenging test implied that superoxide radicals and holes played a significant role in the degradation of orange G, while the contribution of hydroxyl radicals was relatively less (Fig. S12a†). The photodegradation performance of S6 was assessed for 60 min under various wavelength ranges of light (Fig. S12b†). Under direct sunlight (740 W m^−2^), as a source for both visible and NIR light, 100% orange G degradation was achieved. When using sunlight along with an NIR pass filter (*λ* ≥ 720 nm, intensity = 185 W m^−2^), 21% of the dye was degraded. However, under 50 W COB-LED (a pure visible-light source) with a measured illumination intensity of 504 W m^−2^ comparable to that of direct sunlight, only 11% degradation was observed. This unusual result suggests that the photocatalytic mechanism of S6 relied on a laddered heterojunction and required both visible and NIR light for enhanced activity. As a result, S6 performed exceptionally well under direct sunlight due to its ability to utilize a broad spectrum of visible and NIR light.

**Fig. 10:**
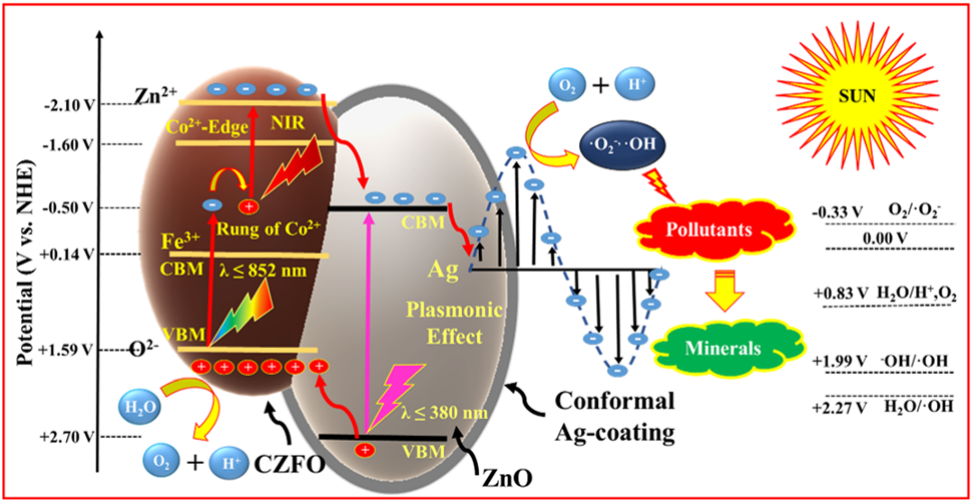
Schematic of the mechanism of pollutant degradation over S6 under direct sunlight.

On the basis of these facts and observations, we propose the following mechanism. When the S6 dispersion in polluted water is exposed to sunlight, the valence band and ground-state conduction band electrons of CZFO get excited to the CB and Zn^2+^ levels, respectively (Fig. 10). The excited CB electrons get transferred to ZnO by the internal electric field (directed from ZnO to CZFO), while valence band electrons trapped by the holes are generated in the conduction band by the excited CB electrons. These electrons further get excited to the Zn^2+^ level and then transfer to the ZnO, leaving holes in the conduction band that trap the next exciting electrons from the VB. This results in a laddered heterojunction, in which electron transfer to ZnO is aided by ladder-like transitions caused by the ladder-rung-like behavior of the Co^2+^ states. Further, the formed Schottky barrier at the ZnO/Ag interface traps the electrons in Ag from ZnO. These electrons due to the LSPR behavior of the Ag coating acquire a suitable potential to react with O_2_ and produce an enormous amount of superoxide radicals, as indicated by the confirmatory and scavenging tests. These generate superoxide radicals, resulting in the decomposition of pollutants, as well as highly reactive hydroxyl radicals that take part in the degradation. Hence, the photocatalyst S6 can efficiently harvest solar energy and transform this into suitable chemical energy for the degradation of various organic pollutants under direct sunlight.

## Conclusions

In the current work, we synthesized a Co^2+^-based laddered heterojunction between Co^2+^-doped zinc ferrite and zinc oxide using co-precipitation and microwave-assisted routes. These heterojunctions were coated with a small fraction of Ag, conformally and selectively, on the surface of ZnO using a facile sunlight-assisted photocatalytic silver reduction method. The resulting photocatalyst could absorb the full spectrum of sunlight, ranging from ultraviolet to near-infrared light. Our studies under direct sunlight, using orange G as a model dye, yielded the following observations described below.

The composite obtained *via* the co-precipitation method, at an optimum temperature of 150 °C for 4 h, photodegraded orange G with an apparent rate constant of 0.012976 min^−1^. The as-prepared composite photocatalyst synthesized by the microwave-assisted route outperformed that prepared by the co-precipitation method. The highest photodegradation was achieved using the catalyst prepared at an annealing temperature of 350 °C (*k* = 0.03074 min^−1^). As a result of plasmonic enhancement by the sharp structure of the ZnO sheets, the conformal Ag coating selectively applied on ZnO further improved the activity, with an optimal Ag content of 2.5%, showing the highest photodegradation rate (*k* = 0.06413 min^−1^). Studies with varied amounts of CZFO and ZnO while keeping the Ag content fixed indicated a relative mole percentage ratio of 1 : 12 as the optimum, achieving a photodegradation rate of 0.1116 min^−1^. Sample S6 could effectively degrade high-COD (∼740 ppm) ciprofloxacin solution under direct sunlight without sacrificial chemicals. Within 60 min, 66% of the ciprofloxacin COD was eliminated. The photocatalyst, without sacrificial chemicals, rapidly decomposed various other dyes too (crystal violet, rose bengal, malachite green, rhodamine B, methyl orange, fluorescein sodium salt, and orange G) and pharmaceuticals (50 μM ciprofloxacin). More than 80% decomposition occurred within 2.5–20 minutes, with complete elimination within 20–60 min under direct sunlight. In conclusion, the prepared photocatalyst holds promise for solar photocatalysis, allowing harnessing the maximum sunlight energy to combat water pollution and enable solar-energy conversion. Its potential applications span across various industries and societal applications, addressing environmental challenges and advocating for sustainable and responsible practices.

## Experimental

### Materials

Iron(iii) nitrate nonahydrate (≥98%, Merck), zinc(ii) nitrate hexahydrate (≥96%, Merck), zinc(ii) acetate dihydrate (Merck), cobalt(ii) nitrate hexahydrate (≥98%, Merck), sodium acetate trihydrate (≥98.5%, Merck), polyethylene glycol 1500 (PEG, Merck), sodium hydroxide (≥97%, Merck), ethylene glycol (EMPLURA®, Merck), and silver nitrate (Merck) were used in the synthesis of the photocatalyst. Deionized water (18 MΩ cm), orange G, ciprofloxacin tablets, terephthalic acid (TA) (Loba), nitro blue tetrazolium chloride (NBT) (≥98%, Loba), *p*-benzoquinone (≥98%, Sigma), *tert*-butyl alcohol (EMPLURA®, Merk), and potassium iodide (Assay 99.4%, Sigma) were used in the photodegradation studies. Potassium hydrogen phthalate (Merck), sulfuric acid (Merck), potassium dichromate, mercury sulfate, and silver sulfate, were used to determine the chemical oxygen demand.

### Synthesis of Co^2+^-doped ZnFe_2_O_4_ (CZFO) nanospheres

The microwave-assisted solvothermal technique (MAST), as reported in ref. 24, was employed for the CZFO synthesis.

### ZnO preparation and annealing of the CZFO/ZnO composite

The synthesis of the CZFO/ZnO composite was carried out by two methods, namely (a) co-precipitation and (b) microwave-assisted reflux methods.

#### Co-precipitation method

First, Zn(OH)_2_ nanoparticles were prepared by the co-precipitation method. Here, 4.38 g of Zn(NO_3_)_2_·6H_2_O was dissolved in 80 ml of DI water. To this solution, 1 M NH_4_OH was added dropwise under vigorous stirring until the pH reached 8.2. The obtained white precipitate was vacuum filtered, washed with DI water, and then dried in a desiccator at room temperature. In order to synthesize the composite, two colloidal dispersions were prepared: A: the as-prepared zinc hydroxide in 120 ml DI water, and B: 600 mg of CZFO in 100 ml DI water. Dispersion B was added to dispersion A and the pH of the dispersion was adjusted to 8.2 using 1 M ammonia solution under vigorous stirring. The precipitate was separated, washed, and then dried in an oven at 80 °C. Finally, samples were annealed at 130 °C, 150 °C, 170 °C, and 200 °C.

#### Synthesis of CZFO/ZnO composite by the microwave-assisted reflux method

The CZFO/ZnO composite was synthesized in various mole ratios of CZFO relative to ZnO, namely 1 : 4, 1 : 6, 1 : 9, 1 : 12, 1 : 15, by a microwave-assisted reflux method. For the synthesis of the composite in the ratio of 1 : 6; two solutions were prepared: Solution A: 1.62 g of zinc(ii) acetate dihydrate was dissolved in 70 ml of DI water. In this solution, 300 mg of CZFO was dispersed; Solution B: 1200 mg of NaOH was dissolved in 30 ml of DI water. Solution B was added to the dispersion in solution A dropwise under vigorous stirring for 20 min and the dispersion pH was raised between 12 and 13. The final solution was transferred to a 500 ml round-bottom flask and irradiated with microwaves for 30 min in a domestic microwave oven (LG, 800 W). Various other composite ratios were formed by varying the amount of zinc(ii) acetate dihydrate and NaOH, in the order shown in Table 2.

**Table 2:** Quantity of salts varied for the synthesis of the CZFO/ZnO composite

CZFO : ZnO	Amount of Zn(NO)_3_·2H_2_O (g)	Amount of NaOH (g)
1 : 6	1.62	1.2
1 : 9	2.43	1.8
1 : 12	3.24	2.4
1 : 15	4.05	3

### Ag coating on Co^2+^-laddered heterojunction

In a 100 ml beaker, the following amounts of CZFO/ZnO given in Table 3 were dispersed in 70 ml silver nitrate aqueous solution. The dispersion was then transferred to a 500 ml beaker and kept under sunlight for an hour between 12 pm and 1 pm, then the samples were centrifuged, washed, and dried in an oven at 80 °C.

**Table 3:** Quantity of CZFO/ZnO composite and AgNO_3_ used during Ag coating

% Ag targeted	Amount of AgNO_3_ (mg) added	Amount of CZFO/ZnO (mg)
2	3.8	116.2
2.5	4.8	115.2
3	5.8	114.2
3.5	6.7	113.3

### Characterizations

The crystallinity of the synthesized materials was characterized by powder X-ray diffraction (XRD, Rigaku Ultima IV, Cu K_α_ radiation) at a scanning rate of 0.5° min^−1^ in the 2*θ* range of 25°–75°. Morphological investigation of the samples was carried out by field emission scanning electron microscopy (FESEM, ZEISS ULTRA-55). The diffuse reflectance spectra of the samples were collected in a UV/Vis/NIR spectrophotometer (PerkinElmer Lambda 750). Nitrogen sorption measurements (Belsorb Mini (II), BEL, Japan) were used to determine the specific surface area, pore volume, and size of the nanoparticles. Nitrogen adsorption–desorption isotherms were measured at 77 K after degassing the samples at 10^−2^ kPa and 120 °C for 3 h. Photoluminescence spectra were collected using a fluorescence spectrometer equipped with a 150 W xenon lamp and a high-speed chopper (Jasco FP8300). The absorption spectra of the aliquots were collected using a UV-Vis spectrophotometer (V-650, JASCO, UK).

### Photocatalytic studies under direct sunlight

#### Optimization of the photocatalytic activity under direct sunlight

Orange G dye solution at 0.05 mM concentration was chosen as a model organic pollutant to study the photodegradation activity of the samples. Prior to the photodegradation studies, 50 mg of the sample was dispersed in 50 ml of orange G solution and kept in the dark for 90 min to establish adsorption–desorption equilibrium of the dye over the photocatalyst samples. Soon after confirming the equilibrium by monitoring the dye concentration at 30 min intervals, the dispersion was exposed to sunlight (Average intensity was 740 W m^−2^) between 11:45 am to 12:45 pm. The photocatalytic experiments were carried between January to April at Manipal, India (GPS co-ordinates 13.353046, 74.793905). For the desired time interval, 2 ml of the aliquot was sampled and centrifuged at 10 000 rpm for 10 min to separate the photocatalyst particles from the dye solution. The absorption spectra of the dye solution in the desired time interval were collected and the instantaneous concentration was monitored.

#### Photocatalytic degradation of ciprofloxacin under direct sunlight

Ciprofloxacin tablets, which were purchased from a local medical store, were used as the source for the antibiotic drug. In a typical 50 ml of 25 ppm ciprofloxacin tablet solution, about 50 mg of the photocatalyst was dispersed and aged in the dark for 90 min and then exposed to sunlight. Aliquots were collected at time intervals of 5, 10, 15, and 20 min of sunlight exposure, and UV-visible absorption spectra were collected by separating the solid particles by centrifugation at 10 000 rpm for 10 min.

#### Degradation of highly concentrated pollutants

In 50 ml of around 400 ppm of ciprofloxacin solution in four separate beakers, respectively, 25, 50, 75, and 100 mg of the photocatalyst were dispersed, and the solutions were then aged in the dark for 90 min, and then exposed to sunlight for 60 min. Soon after the exposure, the photocatalyst was separated by centrifugation and the chemical oxygen demand (COD) of the solution before and after the photocatalysis was determined by the standard available colorimetric method. To determine the change in COD for the different times of exposure, 75 mg of the photocatalyst was dispersed in 50 ml of 400 ppm ciprofloxacin solution and exposed to direct sunlight. At time intervals of 0, 2.5, 5, 10, 20, and 30 min, 2.5 ml aliquots were collected and the corresponding COD values were determined.

#### Degradation of various dyes

In 50 ml separate solutions of crystal violet (20 μM), rose bengal (40 μM), malachite green (100 μM), rhodamine B (15 μM), methyl orange (50 μM), fluorescein sodium salt (30 μM), and orange G (50 μM) 75 mg of S6 was dispersed and the solutions were then aged in the dark for 90 min. After adsorption–desorption equilibrium was reached, the dispersions were exposed to direct sunlight and the concentration of the dye at the desired time interval was monitored.

### Reusability test

To test the photocatalyst for its reusability, each 50 mg sample was dispersed in two beakers both containing 50 ml of 0.05 mM orange G solution and exposed to sunlight soon after adsorption–desorption equilibrium was reached. The photocatalyst was separated by centrifugation soon after the sunlight exposure and dried in an oven at 80 °C for 12 h. The dispersion in one beaker was used to monitor the concentration of the dye before and after 60 min sunlight exposure, whereas the other was used to compensate for the lost photocatalyst during the separation process.

### Confirmatory test for the hydroxyl radicals

Hydroxyl radical generation was confirmed with the help of terephthalic acid as a fluorescent probe. About 166 mg of terephthalic acid was dissolved in 50 ml of 0.05 M NaOH solution. To this solution, 50 mg of the samples were dispersed with the aid of ultrasonication for 3 min and the solutions were then kept in the dark for 60 min. The dispersions were then exposed to sunlight for 5 min, followed by collection of 2 ml of the aliquots, and the photocatalyst was separated out from the solution by centrifugation. The emission spectra of the solution before and after the sunlight exposure was collected at the excitation wavelength of 315 nm.

### Confirmatory test for the superoxide radicals

In order to confirm the generation of superoxide radicals, about 50 mg of the photocatalyst was dispersed in 0.025 mM NBT solution and this solution was then kept in the dark for 60 min. Soon after this, the dispersion was exposed to sunlight and a 2 ml aliquot was collected in the subsequent time interval. After separating the photocatalyst from the aliquot, the absorption spectra were recorded.

### Scavenging test

The scavenging test was conducted using *tert*-butyl alcohol (TBA) as a hydroxyl radical scavenger, *p*-benzoquinone as a superoxide radical scavenger, and KI as a hole scavenger. The photocatalyst was dispersed in 0.05 mM orange G solution in four beakers with or without 2.5 mM of the respective scavengers and then kept in the dark for 90 min. Soon after the establishment of adsorption–desorption equilibrium, the dispersions were exposed to sunlight for 60 min and the absorption of the solution before and after the photocatalysis was measured.

## Data availability

The data supporting this article have been included as part of the ESI.†

## Author contributions

Antony Dasint Lopis: conceptualization, investigation, data curation, methods, writing original draft. Karan Menon: data curation, methods. K. S. Choudhari: methods, review and editing, Bhavana Kulkarni: data curation, methods. Sanjeev P. Maradur, data curation, methods, resources. Suresh D. Kulkarni: conceptualization, investigation, methods, funding, resources, review and editing, supervision.

## Conflicts of interest

Indian patent (no. 427406) has been granted for this study. The authors declare that they have no known competing financial interests or personal relationships that could have appeared to influence the work reported in this paper.

## Supplementary Material

NA-OLF-D4NA00949E-s001
