# Supplementary material for: Solar-driven plasmon-enhanced photocatalysis: Co2+-doped ZnFe2O4 nanospheres-embedded ZnO nanosheets for effective degradation of dyes and antibiotics
Source: Nanoscale Adv. 2025 Jan 15;7(6):1727–35. doi: 10.1039/d4na00949e (PMC11788670; doi:10.1039/d4na00949e)
Supplement: NA-007-D4NA00949E-s001 [file NA-007-D4NA00949E-s001.pdf]

Supplementary Information (SI) for Nanoscale Advances.
This journal is © The Royal Society of Chemistry 2025

Solar-Driven Plasmon-Enhanced Photocatalysis: Co^{2+}-doped $ZnFe_2O_4$ Nanospheres embedded ZnO Nanosheets for Effective Degradation of Dyes and Antibiotics

Antony Dasint Lopis[a], Karan Menon[a], K. S. Choudhari[a], Bhavana Kulkarni[b], Sanjeev P. Maradur[b], Suresh D. Kulkarni[a]*

a Department of Atomic and Molecular Physics, Manipal Academy of Higher Education, Manipal, Karnataka, India -576104

b Materials Science & Catalysis Division, Poornaprajna Institute of Scientific Research (PPISR), Bidalur Post, Devanahalli, Bengaluru, Karnataka, India-562164.

Characterizations

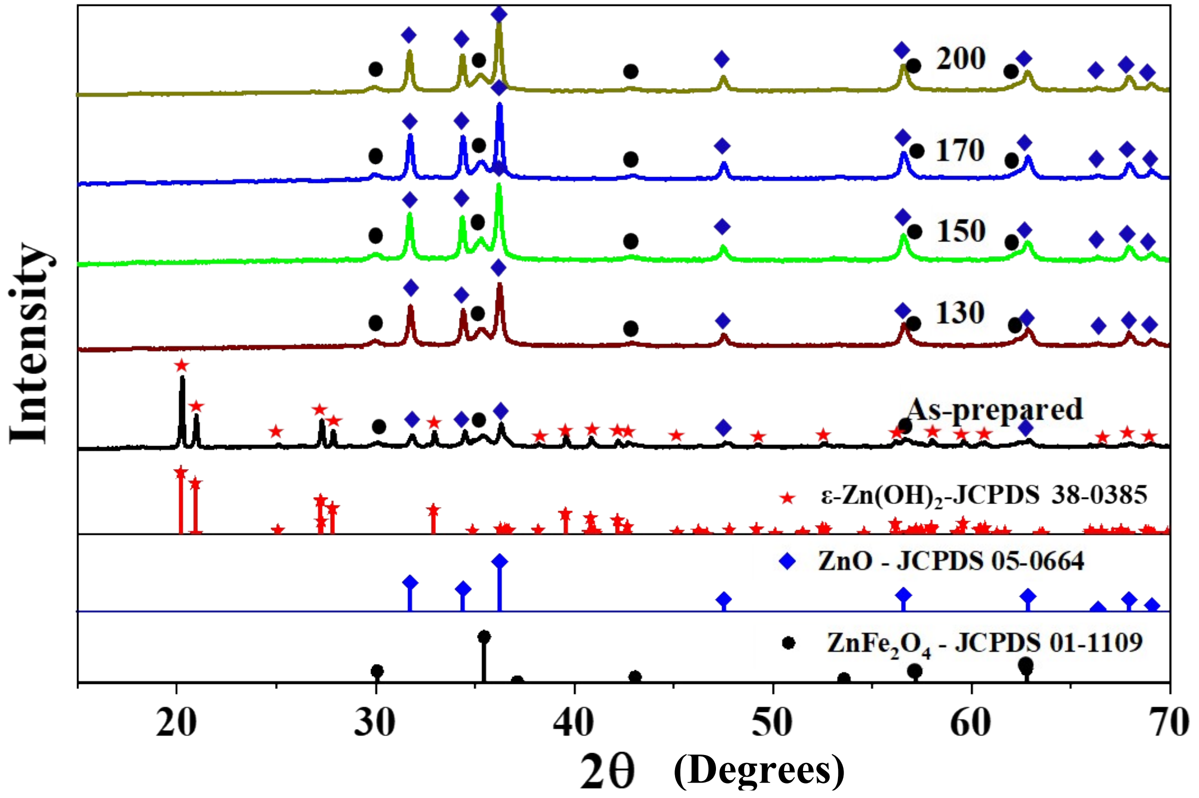

Figure S1 *XRD patterns of as-prepared and annealed CZFO/ZnO composite at 130 °C, 150 °C, 170 °C, and 200 °C for 4h obtained via coprecipitation method*

The XRD patterns of the sample prepared using the coprecipitation method (*Figure S1*) revealed peaks corresponding to hexagonal zinc oxide (JCPDS – 05-0664), cubic spinel zinc ferrite (JCPDS – 01-1109) [1], and an intermediate phase corresponding to Wulfingite $Zn(OH)_2$ (JCPDS 38-0385)[2]. The presence of $Zn(OH)_2$ indicated that the CZFO/ZnO composite formation was incomplete. After annealing at 130 °C, 150 °C, 170 °C, and 200 °C for 4 hours, the zinc hydroxide phase was eliminated. As a result, the ZnO peaks intensified.

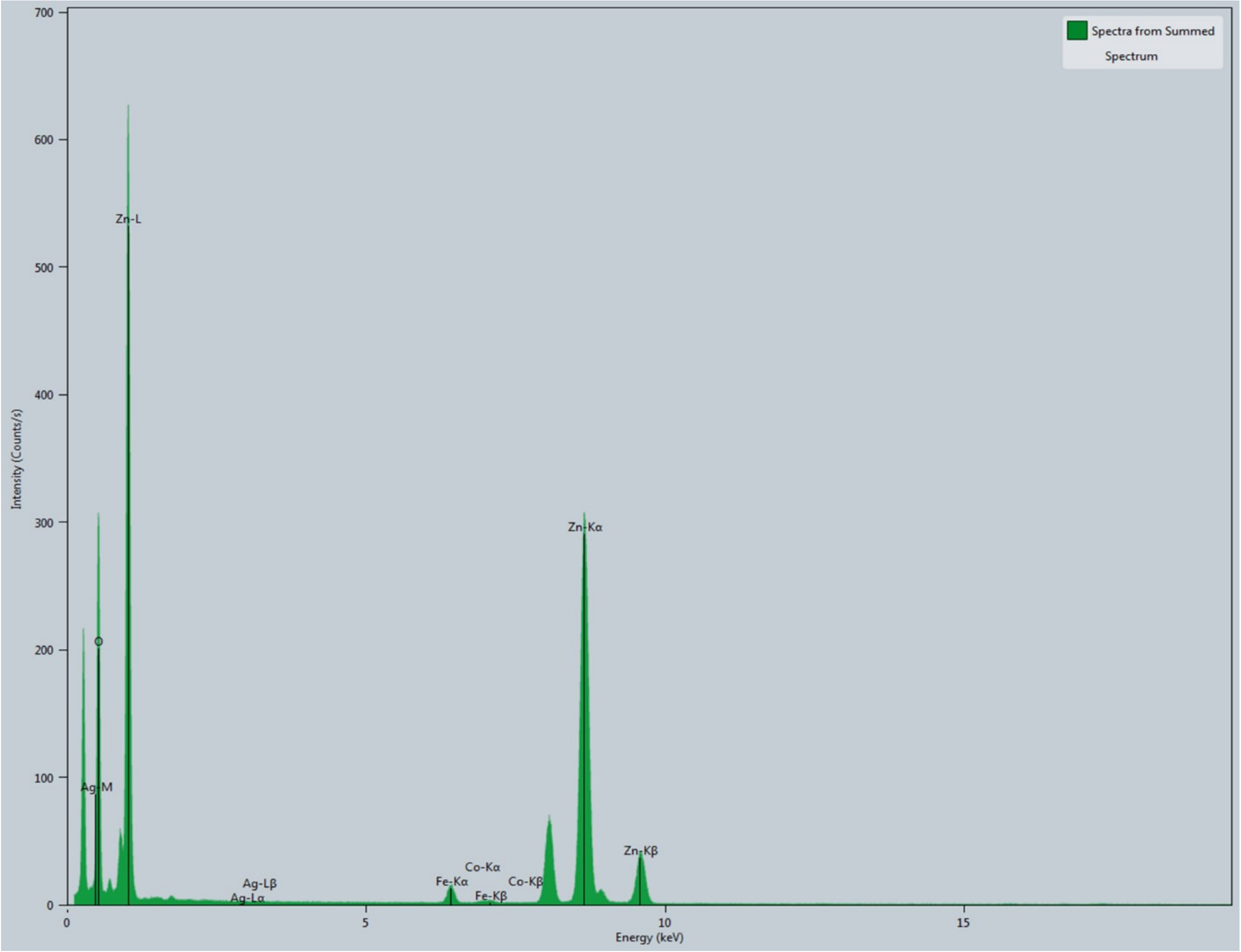

Figure S2 *EDS spectrum of S6,*

Table S1 *BET surface analysis parameters*

Surface properties	Before Ag deposition (S5)	After Ag deposition (S6)
Monolayer volume (cm^3 (STP) g^{-1})	8.1	7.7
Specific surface area (m^2 g^{-1})	35	34
Mean pore diameter (nm)	34.9	33.9
Total pore volume (cm^3 g^{-1})	0.30	0.28

Photodegradation studies of Orange G under direct sunlight

CZFO/ZnO composite obtained from coprecipitation method

The photodegradation of Orange G dye under direct sunlight was studied using CZFO/ZnO composites obtained by co-precipitation. The study included both as-prepared and annealed samples at various temperatures (130 °C, 150 °C, 170 °C, and 200 °C) for 4 hours, as shown in *Figure S3*. The as-prepared composite exhibited a lower photodegradation rate (k = 0.00591 min-1) due to incomplete formation, as indicated by the XRD analysis (*Figure S1*). However, when annealed to different temperatures the photocatalytic rate was significantly improved. At an optimum temperature of 150° C (sample S1) the highest photocatalytic rate (k = 0.012976 min^{-1}) was observed. The observed photocatalytic rate for different annealing temperatures can be correlated to the decrease in emission intensity (*Figure S4a*). This decrease is attributed to the filling of oxygen vacancies in ZnO of the CZFO/ZnO composite which led to an increase in the rate of degradation. The formation of ZnO with very few oxygen vacancies could be the cause of the lowest emission intensity, and, consequently, the maximum degradation rate for S1. The decrease in photodegradation rate for annealing at temperatures higher than 150° C suggests that annealing at higher temperatures is not beneficial for ZnO formation with minimum defects for the improved photocatalytic activity.

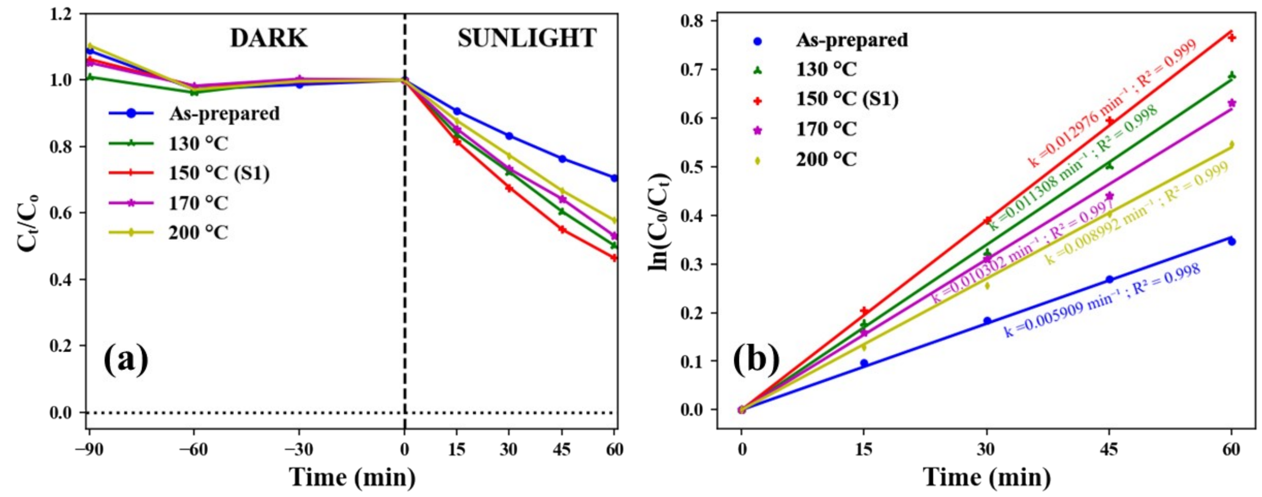

Figure S3 *(a) Plots of the relative concentration of Orange G during the different intervals of time under dark and sunlight in the presence of as-prepared and annealed CZFO/ZnO composite at 130 °C, 150 °C, 170 °C, and 200 °C for 4h obtained via coprecipitation, (b) respective apparent first-order kinetic plots*

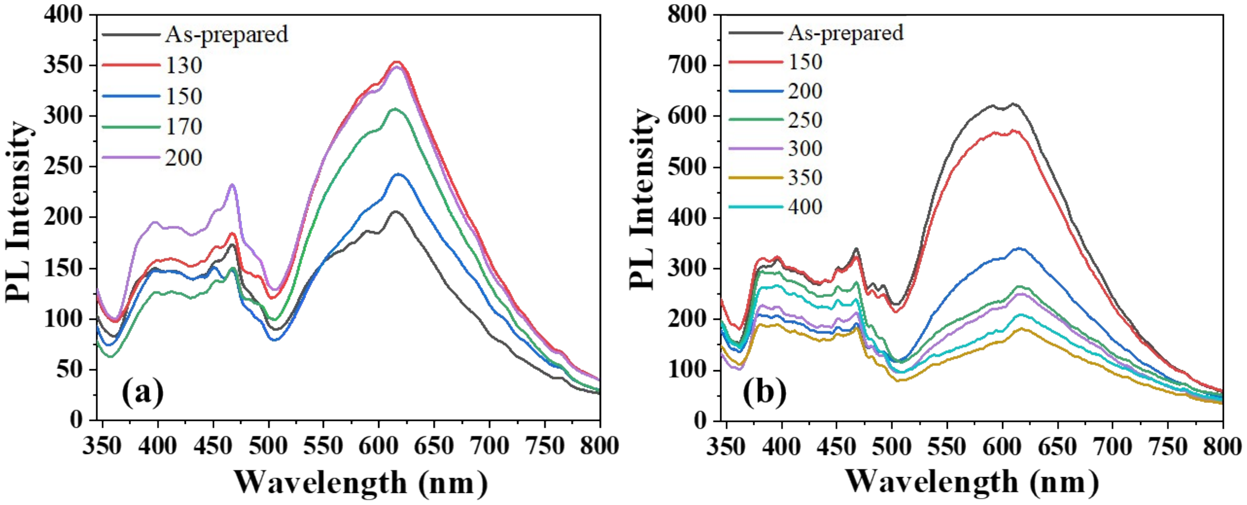

Figure S4 *Emission spectra of (a) as-prepared and annealed CZFO/ZnO composite at 130 °C, 150 °C, 170 °C, and 200 °C for 4h obtained via coprecipitation, (b) as-prepared and annealed CZFO/ZnO composite at 150 °C, 200 °C, 250 °C, 300 °C, 350 °C and 400 °C for 4h obtained via microwave-assisted reflux method.*

CZFO/ZnO composite obtained from microwave-assisted reflux method

The as-prepared CZFO/ZnO composite (S2), was synthesized using the microwave-assisted reflux method, showed an enhanced photodegradation rate (k = 0.01554 min^{-1}) compared to S1. The activity increased as the annealing temperature was raised up to 350 °C (S3).

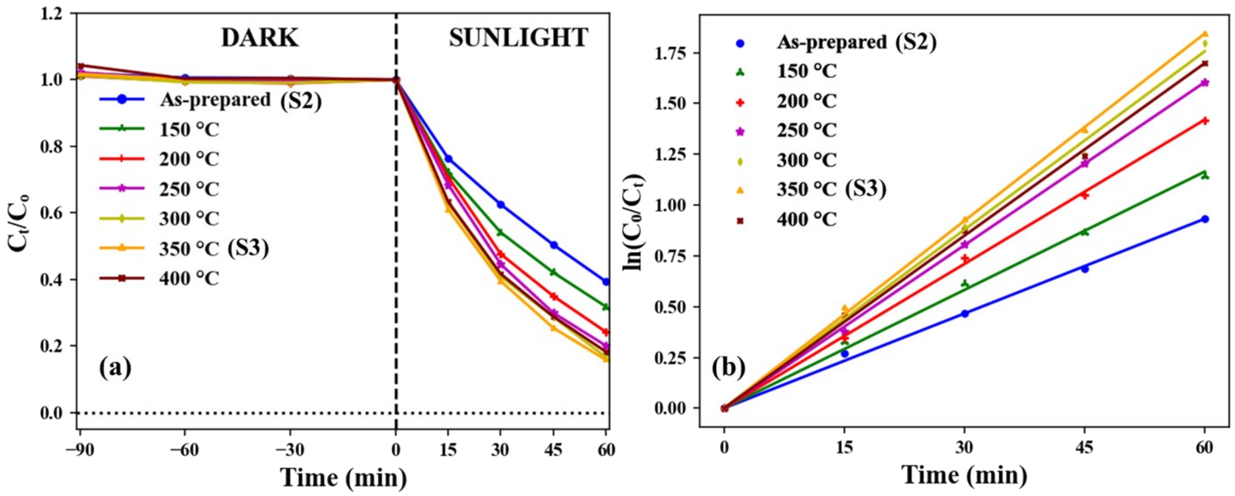

Figure S5 *(a) Plots of the relative concentration of Orange G during the different intervals of time under dark and sunlight in the presence of as-prepared and annealed CZFO/ZnO composite at 150 °C, 200 °C, 250 °C, and 300 °C, 350 °C, and 400 °C for 4h obtained via microwave-assisted reflux method, (b) respective apparent first-order kinetic plots*

As shown in *Figure S5*, at the optimum temperature, the photodegradation rate was 0.03074 min^{-1}. In comparison to S1 and S2, the photodegradation rate for S3 was 2.4 and 2.0 times higher, respectively. The activity decreased (0.0283 min^{-1}) when the annealing temperature was raised further to 400 °C, indicating that 350 °C was the optimal annealing temperature for the best activity (*Table S2*). The observed trend in S2's photocatalytic activity with respect to annealing temperature correlated with the oxygen vacancy-related emission (*Figure S4b*). Consequently, the composite S3 exhibited higher activity in comparison to the other materials, as it contained relatively fewer oxygen vacancies than other annealed samples.

Table S2 *Variation of apparent first-order rate constant of the degradation reaction with photocatalyst annealing temperature*

Annealing temperature (°C)	Apparent first order rate constant	
	k (in min $^{-1}$)	R^2
As-prepared	0.01554	0.9971
150	0.01941	0.9966
200	0.02367	0.9990
250	0.02675	0.9996
300	0.02928	0.9962
350	0.03074	0.9992
400	0.02830	0.9985

Effect of Ag-deposition

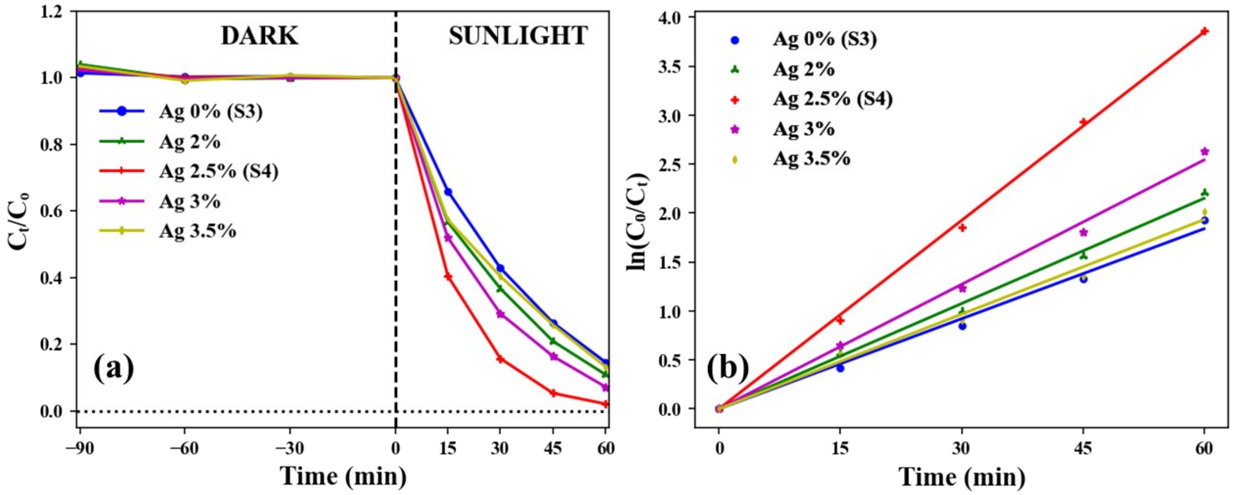

Figure S6 *(a) Plots of the relative concentration of Orange G during the different intervals of time under dark and sunlight in the presence of S3 and S3 coated with 2%, 2.5%, 3%, and 3.5% of Ag, (b) respective apparent first-order kinetic plots.*

As depicted in Figure S6a, S3's photodegradation activity improved following Ag deposition due to plasmon-based enhancement. The photodegradation rate doubled with an increase in Ag content until reaching the optimal percentage of about 2.5% (referred to as sample S4). Beyond this threshold, the rate decreased (*Table S3*). These findings indicate that the LSPR behavior of the Ag coating plays a crucial role in enhancing and optimizing the catalytic activity. The absorption based on LSPR is generally influenced by the quantity of Ag, as well as its shape and size. It is evident that 2.5% of Ag produces a stronger LSPR effect on S3, resulting in the highest photocatalytic activity.

Table S3 *Variation of apparent first-order rate constant of the degradation reaction with different amounts of Ag deposition*

Weight percent of Ag (%)	Apparent first order rate constant	
	k (in min $^{-1}$)	R^2
0.0	0.03065	0.9952
2.0	0.03582	0.9957
2.5	0.06413	0.9988
3.0	0.04235	0.9953
3.5	0.03220	0.9891

Effect of different amounts of ZnO loading

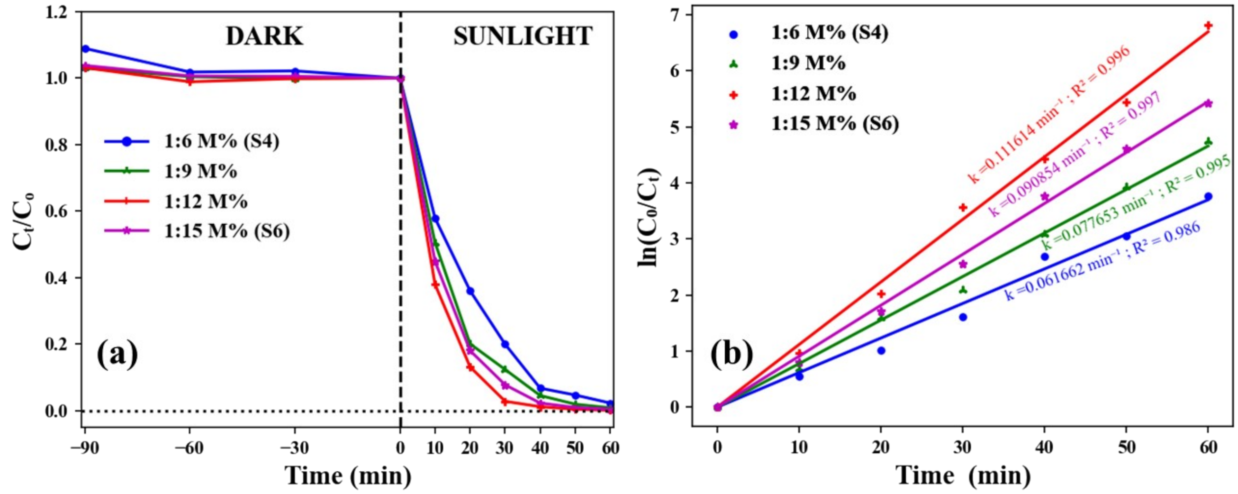

Figure S7 *(a) Plots of the relative concentration of Orange G during the different intervals of time under dark and sunlight in the presence of CZFO/ZnO/Ag composite obtained via microwave-assisted reflux method with different ratios of CZFO: ZnO (b) respective apparent first-order kinetic plots*

As depicted in *Figure S7*, the degradation rate increased from 0.0617 min^{-1} to 0.1116 min^{-1} as the ZnO content increased from CZFO:ZnO: 1:6 to 1:12.Further increase in ZnO content led to a decrease in the rate to 0.09085 min^{-1}. This observation can be attributed to the improved junction potential between CZFO and Ag as a result of increased ZnO content, leading to greater charge separation. After surpassing the optimal level, the reduced CZFO content led to decreased sunlight absorption, resulting in a lower photocatalytic rate. Therefore, the most effective composition for achieving the highest activity was a ratio of 1 part ZnO to 12 parts CZFO. The

samples containing CZFO and ZnO in a 1:12 ratio, before and after 2.5% Ag deposition, are denoted as S5 and S6, respectively.

The Orange G dye offers good light fastness, as demonstrated by independent photodegradation studies (*Figure S8*). The blank dye showed no change in concentration when exposed to sunlight. In presence of CZFO alone there was negligible degradation (<10%) indicating the need for ZnO to have significant photocatalytic activity. In the absence of Ag, S5 degraded Orange G at a rate of 0.0565 min^{-1} , and the rate constant doubled in its value for S6 (0.1121 min^{-1}), highlighting the contribution of Ag to the augmentation of the photocatalytic activity.

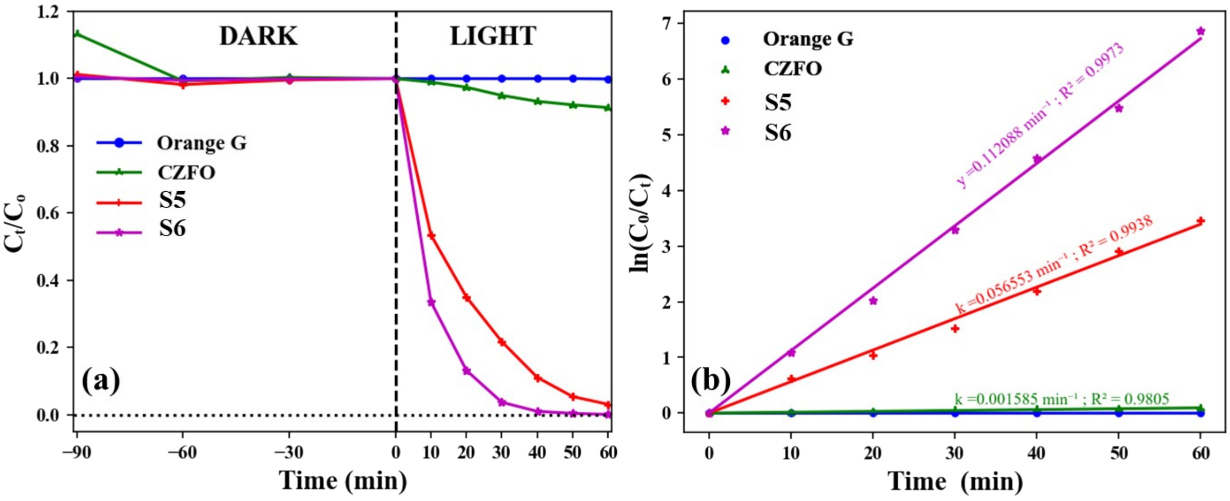

Figure S8 *(a) Plots of the relative concentration of Orange G during the different intervals of time under dark and sunlight in the absence and presence of CZFO, S5, S6 (b) respective apparent first-order kinetic plots*

The absorption spectra of the Orange G dye were measured at various time intervals while exposed to sunlight in the presence of S6. The results showed a reduction in absorbance over time (*Figure S9a*). When Orange G was aged in the presence of S6 in the dark for 90 minutes, there was only a modest variation in its absorbance. However, when exposed to sunlight, the absorbance drastically dropped within 10 minutes, indicating rapid photo-degradation.

Reusability test

Given the enormous amounts of pollutants produced by industries, it is preferred that the photocatalyst be to be inexpensive and capable of being reused multiple times without losing its effectiveness. To test this, the photocatalyst S6 was extracted from the degraded solution and reused to decompose a fresh dye solution. It was observed that the percentage of degradation did not change significantly after five cycles of reuse, with only a 2% decrease in activity (*Figure S9b*). This suggests that the photocatalyst can be potentially reused several times.

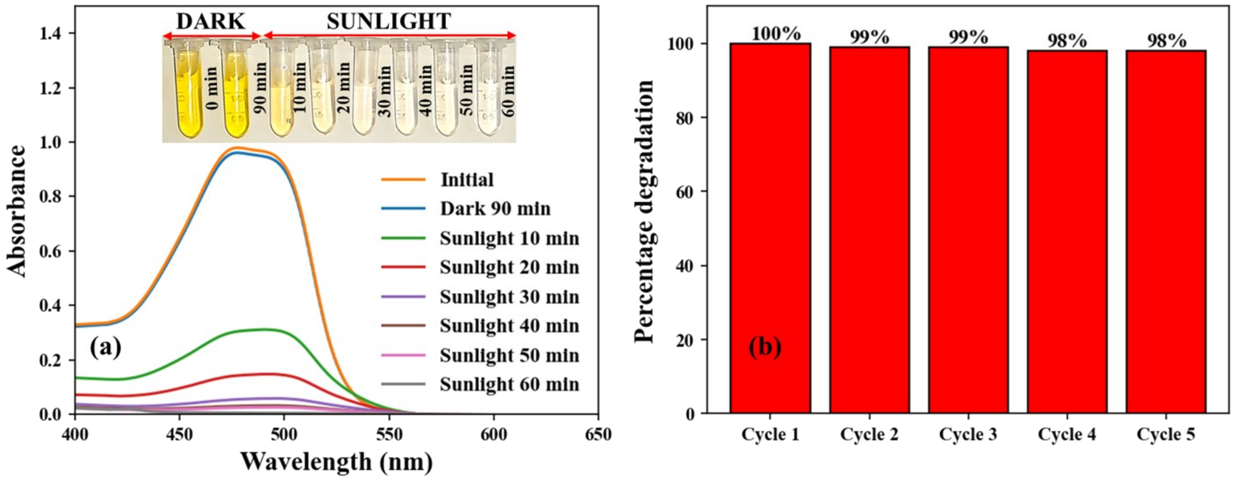

Figure S9 *(a) Absorbance spectra of Orange G dye at the different time intervals of photocatalysis, (b) the percentage of Orange G degradation for five successive photocatalytic runs of S6.*

Table S4 *XRD pattern deconvolution results of S6 before and after 5-cycles of reuse*

	$\frac{ZnO\ (101)}{ZnFe_2O_4(311)}$	$\frac{Ag\ (111)}{ZnO\ (101)}$	Ag average crystallite size (nm)	R^2
Before	2.80	0.092	34	0.9977
After	2.78	0.095	34	0.9981

Photocatalytic degradation of various dyes

The photodegradation capability of S6 was tested with various dyes using an optimum catalyst concentration of 1.5g/L (*Figure S10*). The target dyes and their respective concentrations were as follows: Crystal Violet (20 µM), Rose Bengal (40 µM), Malachite Green (100 µM), Rhodamine B (15 µM), Methyl Orange (50 µM), Fluorescein Sodium Salt (30 µM), and Orange G (50 µM) (*Table S5*). S6 demonstrated excellent photodegradation capability under sunlight. It eliminated

more than 80% of Malachite Green, Rhodamine B, and Fluorescein Sodium Salt within 2.5 minutes and Crystal Violet within 10 minutes. Complete degradation of these dyes was observed within 20 minutes, while Orange G and Methyl Orange degraded in 40 minutes. Rose Bengal degraded in 60 minutes under sunlight.

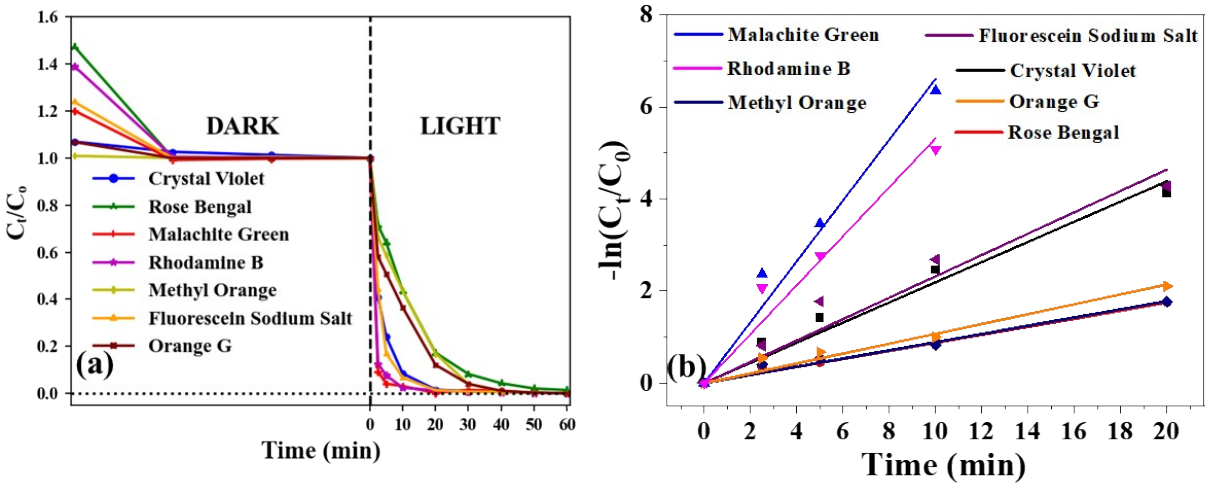

Figure S10 *(a) Degradation kinetics of Crystal Violet (20 µM), Rose Bengal (40 µM), Malachite Green (100 µM), Rhodamine B (15 µM), Methyl Orange (50 µM), Fluorescein Sodium Salt (30 µM), and Orange G (50 µM) over S6 under direct sunlight. (b) Plots for the determination of the* apparent first-order rate constant.

Table S5 *Degradation kinetics monitoring table for different dyes*

Dye targeted	Dye Concentration (µM)	Time for > 80% degradation (min)	Time for 100% degradation (min)	Apparent 1st order rate constant (min^{-1})	R^2
Crystal violet	20	10	20	0.2193	0.9859
Rose Bengal	40	20	60	0.0874	0.9961
Malachite Green	100	2.5	20	0.6616	0.9896
Rhodamine B	15	2.5	20	0.5322	0.9837
Methyl Orange	50	20	40	0.0892	0.9891
Fluorescein Sodium Salt	30	2.5	20	0.2322	0.9763
Orange G	50	20	40	0.1076	0.9836

Confirmatory test for ROS

The NBT and TA were used to respectively probe the generated superoxide radicals and hydroxyl radicals. As shown in *Figure S11*, in the presence of S6 under sunlight, the absorption peak at 260 nm corresponding to NBT reduced to more than 80% within 5 min. As shown in *Figure S11* inset, the dispersion turned dark, indicating the formation of a dark blue-colored insoluble formazan resulting from the reaction of generated superoxide radicals with the NBT. This suggests the formation of a large amount of superoxide radicals over S6 due to the efficient utilization of the full solar spectrum [3, 4].

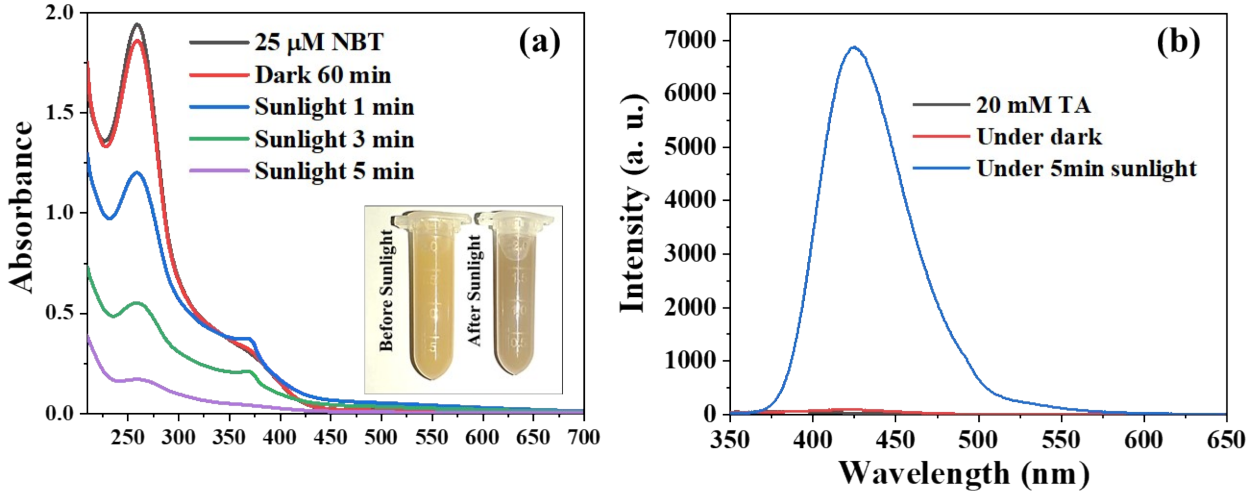

Figure S11 *(a) Absorbance spectra of Ciprofloxacin NBT at different intervals of sunlight exposure in the presence of S6, (b) emission spectra of 20 mM terephthalic acid solution before and after the sunlight exposure in the presence of S6.*

The non-fluorescent TA solution started to fluoresce after 5 minutes of direct sunlight exposure, indicating the formation of hydroxyl radicals and the subsequent production of 2-hydroxyterephthalic acid through the reaction of TA with the generated hydroxyl radicals (*Figure S11b*).

Scavenging Test

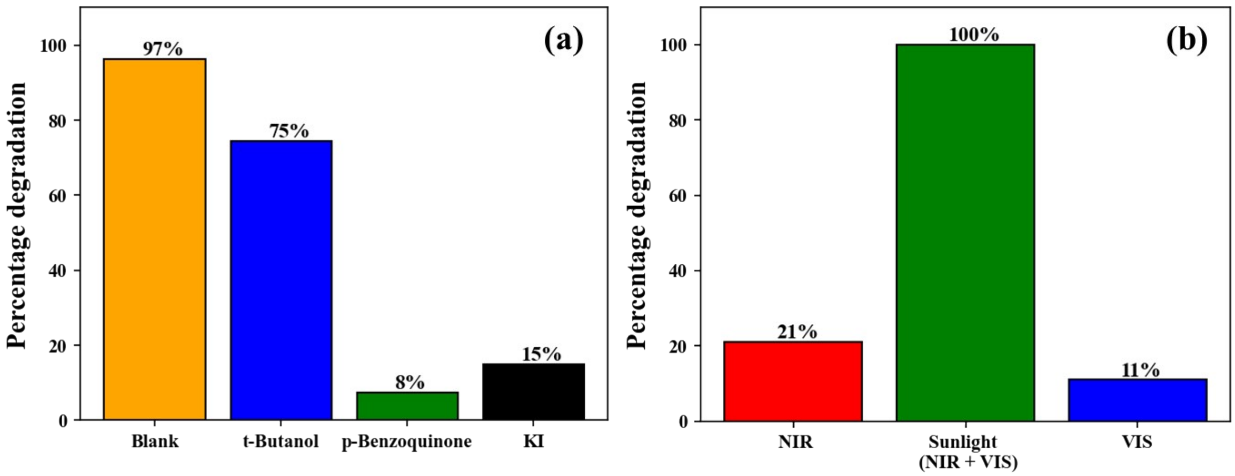

Figure S12 *Percentage of degradation of Orange G under 30 min sunlight in the presence and absence of t-Butanol, p-Benzoquinone, and KI as respective hydroxyl radicals, superoxide radicals, and holes scavenger (b) percentage of Orange G degradation under different light sources for 30 min.*

We, further, investigated the impact of different reactive species on photodegradation, both with and without the presence of scavengers (*Figure S12a*). When no scavengers were present, 97% of Orange G degraded after 30 minutes of sunlight exposure. However, in the presence of p-Benzoquinone (a superoxide scavenger) and KI (a scavenger for holes), only 8% and 15% of the dye degraded, respectively. In the presence of t-Butanol (a hydroxyl radical scavenger), 75% of Orange G degradation was observed. This suggests that superoxide radicals and holes play a significant role in the degradation of Orange G, while the contribution of hydroxyl radicals is relatively less. The reason that superoxide radicals contribute more than hydroxyl radicals stems from the fact that hydroxyl radicals are produced indirectly by superoxide radicals.

References

1. Sai, R.; Kulkarni, S. D.; Vinoy, K. J.; Bhat, N.; Shivashankar, S. A. *Journal of Materials Chemistry* **2012,** 22, (5), 2149-2156.
2. Wang, J.; Hou, S.; Zhang, L.; Chen, J.; Xiang, L. *CrystEngComm* **2014,** 16, (30), 7115-7123.
3. Nosaka, Y.; Nosaka, A. Y. *Chemical Reviews* **2017,** 117, (17), 11302-11336.
4. Shekofteh-Gohari, M.; Habibi-Yangjeh, A.; Abitorabi, M.; Rouhi, A. *Critical Reviews in Environmental Science and Technology* **2018,** 48, (10-12), 806-857.
